# 

**Published:** 1895-04

**Authors:** 


### TABLE OF CONTENTS.

ORIGINAL COMMUNICATIONS. PAGE

Gouty Pericementitis. By Edwin T. Darby, M.D., D.D.S., Philadelphia . 197

Notes on Hydrogen Dioxide ($H_2O_2$). By George S. Allan, D.D.S., New York City . . . . . . . . . . . . . . . . . . . . . . . . . . . . . . 204

Bleaching Teeth by Electricity. By Dr. Albert Westlake, Brooklyn, N. Y. . . . . . . . . . . . . . . . . . . . . . . . . . . . . . . . . 213

Some Principles relating to Amalgams. By Edward C. Kirk, D.D.S., Philadelphia . . . . . . . . . . . . . . . . . . . . . . . . . . . . . 214

REPORTS OF SOCIETY MEETINGS.

New York Odontological Society . . . . . . . . . . . . . . . . . . . . . 223

Academy of Stomatology . . . . . . . . . . . . . . . . . . . . . . . . 240

EDITORIAL.

Specialism in Dentistry . . . . . . . . . . . . . . . . . . . . . . . . 253

Explanation . . . . . . . . . . . . . . . . . . . . . . . . . . . . . 256

The *Dental Digest* . . . . . . . . . . . . . . . . . . . . . . . . . 256

DOMESTIC CORRESPONDENCE.

A New Method of reducing Dislocation of the Jaw . . . . . . . . . . . 257

CURRENT NEWS.

Tri-State Meeting . . . . . . . . . . . . . . . . . . . . . . . . . . 258

Union Meeting . . . . . . . . . . . . . . . . . . . . . . . . . . . . 259

SELECTIONS.

Sulphuric Acid for opening Root-Canals . . . . . . . . . . . . . . . . 259

